# Apoptosis Induced by Metal Complexes and Interaction with Dexamethasone

**DOI:** 10.1155/MBD.2002.249

**Published:** 2002

**Authors:** Jung Sun Kim, José Carlos Almeida Barros, Szulim Ber Zyngier

**Affiliations:** 1 Institute of Biomedical Sciences of the University of São Paulo Department of Pharmacology Av. Prof. Lineu Prestes, 1524 São Paulo SP- 05508-900 Brazil; 2 Faculty of Medical Sciences of the Santa Casa de São Paulo R. Da. Veridiana 311 São Paulo SP-01238-010 Brazil

## Abstract

Apoptosis induced by rhodium II amidate, rhodium II propionate, cisplatin and interactions with dexamethaxone were studied on some human leukemia cell lines Raji, Jurkat and U937. Apoptosis was studied by flow cytometry, agarose gel electrophoresis and morphological analysis. Rhodium II propionate induced apoptosis in all the three cell lines, Rhodium II amidate, in the lymphoid cell lines Jurkat and Raji, and cisplatin, only in the Jurkat, a T lymphoid cell line. It has also been observed that the addition of dexamethasone enhances the apoptosis index only in U937, a monocytic line with a glucocorticoid receptor bearing.


### APOPTOSIS INDUCED BY METAL COMPLEXES AND INTERACTION WITH DEXAMETHASONE

Jung Sun Kim[1], José Carlos Almeida Barros[2], and Szulim Ber Zyngier*[1]

[1]Institute of Biomedical Sciences of the University of São Paulo, Department of Pharmacology, Av. Prof. Lineu Prestes, 1524, São Paulo - SP - 05508-900 –Brazil < szyngier@icb.usp.br>

[2]Faculty of Medical Sciences of the Santa Casa de São Paulo, R. Da. Veridiana, 311 São Paulo, SP - 01238-010, Brazil

### ABSTRACT

Apoptosis induced by rhodium II amidate, rhodium II propionate, cisplatin and interactions with dexamethaxone were studied on some human leukemia cell lines Raji, Jurkat and U937. Apoptosis was studied by flow cytometry, agarose gel electrophoresis and morphological analysis. Rhodium II propionate induced apoptosis in all the three cell lines, Rhodium II amidate, in the lymphoid cell lines Jurkat and Raji, and cisplatin, only in the Jurkat, a T lymphoid cell line. It has also been observed that the addition of dexamethasone enhances the apoptosis index only in U937, a monocytic line with a glucocorticoid receptor bearing.

### INTRODUCTION

For years, it was assumed that radiation therapy and many anticancer drugs killed malignant cells directly harming the DNA of those cells. Now it is known that those treatments just harm the DNA to a minor extent and the affected cells cannot repair the damage and they actively kill themselves. Apoptosis, the programmed cell death, may be triggered by physiological, pathological or pharmacological stimuli. Tumor cells, when exposed to antineoplastic drugs, exhibit apoptosis and alterations of cell cycle phases [1, 2, 3, 4; 5]. These drug effects may have a clinical relevance in acute leukemia patients receiving intensive chemotherapy together with other drugs; for example, effects on acute myeloid (AML) blasts may influence their susceptibility to drug-induced apoptosis, and effects on T cells may alter effector functions that mediate additional antileukemic effects in patients receiving intensive chemotherapy [ 6, ]

Since the initial discovery of the antineoplastic activity of cisplatin several metal complexes including those of rhodium have been tested and some exhibited antitumor activity [7, 8, 9, 10, 11, 12, 13, 14].

Glucocorticoids are known by their limpholytic effects and are used empirically in the treatment of some malignant lymphomas and acute and chronic lymphoblastic leukemias. The antitumor effects of glucocorticoids, including apoptosis, seem to be mediated by binding to a specific cytoplasmatic receptors [15] that are translocated to the nucleus and signal apoptosis. Glucocorticoid-induced apoptosis of lymphoid cells does not require wild-type p53 activity [16].

The aim of this study was to examine the effects of some metal complexes on apoptosis and to disclose interactions by the association of these compounds and the glucocorticoid dexamethasone.

### MATERIALS AND METHODS

Cell lines: The cell lines used in this study have been described previously. Human leukemic cell lines, Raji (B cell lymphoblastic leukemia line) and U937 (monocytic cell line) were obtained from Cells Bank (Rio de Janeiro). Jurkat (T cell lymphoblastic leukemia cell line) was obtained from The University of Oxford. The cell lines were maintained in RPMI 1640 (Sigma) supplemented with 10% fetal calf serum, glutamine and antibiotics. The cultures were incubed at 37° C in a humidified atmosphere with 5% of carbon dioxide.

Drugs: Rhodium II complexes, amidate [14] and propionate, were obtained from the Institute of Chemistry, University of São Paulo. Cisplatin was purchased from Eurofarma Laboratories. Dexamethasone was purchased from BioChimico.

Drug treatment: Approximately $5x10^5$ cells $mL^{-1}$ were exposed to the drugs for 24 hr at $37^{\circ}$ C. Drugs were dissolved in water to the final concentration: 50 μM rhodium (II) amidate; 10 μM rhodium (II) propionate; 200 μM cisplatin; 360 μM dexamethasone. Control cultures received saline.

Flow cytometry: Drug-treated cells were prepared according to the procedure described previously [17]. The DNA content was measured by a Becton Dickinson FACScalibur flow-cytometer and analysed by CellQuest software. The forward scatter (FSC) and side scatter (SSC) of particles were simultaneously measured.

Agarose gel electrophoresis analysis: Cells ($2x10^6$), incubated as above, were lysed in 0,4 mL 10 mM EDTA, 50 mM Tris-HCL, pH8.0, containing 0,5% SDS at $65^{\circ}$ C for 2 hr. The lysate was extracted twice with phenol:chloroform and the DNA precipitated in ethanol. After centrifugation, pellets were dissolved in TE (10 mM Tris-HCL, pH 8.0, 1 mM EDTA) and treated with 0,5 mg/mL RNAse (Sigma) for 3 hr at 37° C. Electrophoresis was carried out at 50 V (constant) in 2% agarose-gel. After electrophoresis, the gel was stained with ethidium bromide (0,5 μg/mL; 15 min) and then destained in water (15 min). Molecular weight markers (100 bp) were purchased from Amersham Pharmacia Biotech.

Morphological studies: The morphology of control and drug-treated cells was studied by staining the cells with the May Grünwald dye [18] . The criteria for the identification of apoptotic features included membrane blebbing, chromatin condensation and formation of apoptotic bodies [19].

Statistical analysis: Resultant data were compared by means of Kruskal-Wallis test with multiple comparisons [20]. When $p \leq .05$, differences were deemed to be statistically significant.

### RESULTS

Rhodium II amidate: Rhodium II amidate induced apoptosis on lymphocitic cell lines, Raji and Jurkat, while U937 cells were resistant. (Figs 1 and 2).

Rhodium II propionate: Rhodium II propionate induced apoptosis on the three cell lines studied (Figs 1 and 2).

Cisplatin: Cisplatin induced apoptosis on Jurkat cells, as seen in Fig 1. This was confirmed by a morphological study of the cells also. The drug did not induce apoptosis on Raji and U937 cell lines.

Dexamethasone: Dexamethasone increased significantly apoptosis on the U937 cell line (also with morphological confirmation).

Association of Dexamethasone with metal complexes: There was no significant difference on the apoptosis index with the association dexamethasone / rhodium II complexes on the cell lines studied. The association cisplatin and dexamethasone did not alter apoptosis on Raji cell lines as well but this association enhanced significantly apoptosis on the U937 cell line and reduced significantly apoptosis on Jurkat cells as compared to cisplatin alone.

### DISCUSSION

In this study we have been observing the apoptosis index in some cell lines induced by metal complexes using direct flow cytometry analysis and agarose electrophoresis (DNA's fragmentation was confirmed by agarose electrophoresis where there was a typical DNA ladder image, indicative of internucleosomal DNA damage). However in some particular situations (enhanced apoptosis rate seen at flow cytometry, with no ladder image at electrophoresis) we need a morphological confirmation. The lack of the DNA's fragmentation ladder on electrophoresis may be due to low rate of apoptotic cells or DNA fragments with high molecular weight [21]. A possible interference of glucocorticoids on those apoptosis rates was also examined.

Rhodium II propionate induced apoptosis in the three cell lines studied. Rhodium II amidate induced apoptosis in both lymphoid cell lines (Jurkat and Raji), while cisplatin induced only in the T lymphoid cell line (Jurkat). Dexamethasone enhanced cell apoptosis only on the monocytic cell line (U937).

The role of apoptosis and acquired mutations have been providing an explanation for the drug sensitivity and resistance in cancer treatments reports. Many oncogens have been found to be related to chemosensitivity and resistance to cytotoxic agents like c-myc, bcl2, mdr and mainly the tumor p53 supressor gene. Mutations of gene p53 are frequent in human leukemic cell lines [22]. Chemotherapeutic drugs may cause apoptosis via p53 mutation [23] or be independent of p53 mutation [24] . The apoptosis induced by the metalic complexes studied here seem to be independent of p53 mutation since Jurkat and U937 cell lines do not express this gen [25] and in Raji cell line this gene is inactivated [26] .

The finding that dexamethasone induces apoptosis in U937 is coherent with the hypothesis that glucocorticoids induce apoptosis mediated by their binding to a specific cytoplasmatic receptor [27] since this cell line has a glucocorticoid receptor [28, 29] while Jurkat cells do not have glucocorticoid receptors [30], and Raji cells have a low glucocorticoid receptor [31, 32].

There were no interactions by association of dexamethasone and rhodium II complexes. Association of dexamethasone with cisplatin enhanced apoptosis in glucocorticoid receptor bearing cells U937 but reduced apoptosis in the T lymphoblastic leukemia cells Jurkat.

### ACKNOWLEDGMENTS

The authors are grateful to Fundação de Amparo à Pesquisa do Estado de São Paulo (FAPESP) for financial support. One of the authors (JSK) is also grateful to the Conselho Nacional de Desenvolvimento Científico e Tecnicológico (CNPq) for a Ph.D. fellowship grant.

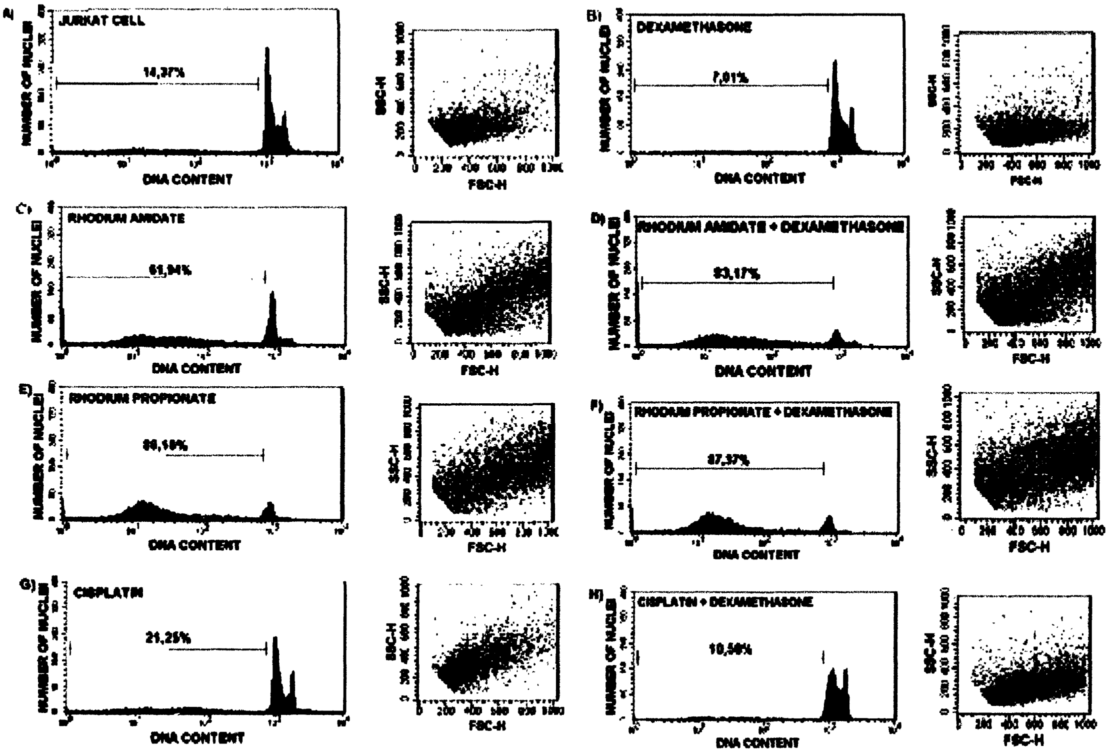

Fig 1 DNA's histogram (log scale) and apoptosis indexes of Jurkat cells submitted to metalic complexes for 24 hours with and without dexametasone. In each sample 10,000 events were analysed through the FACScalibur from Becton Dickson.

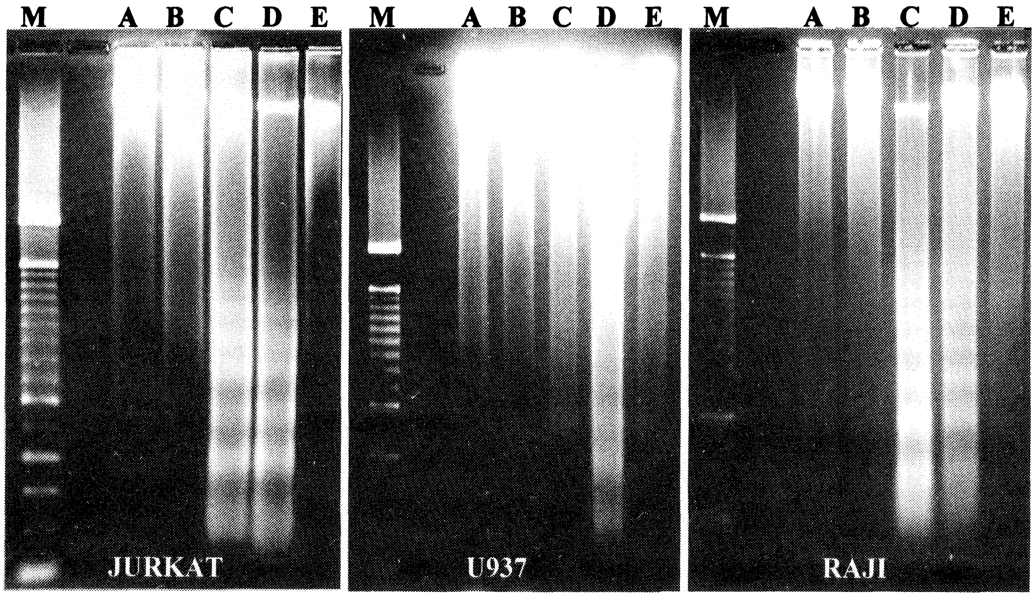

Fig 2: Picture of DNA's gel- agarose electrophoresis of the Jurkat, Raji and U937 cell lines **A** control, **B** dexamethasone, **C** rhodium amidate, **D** rhodium propionate, **E** cisplatin, **M** molecular weight marker of 100bp. Ladder images, indicate internucleosomal DNA damage

### REFERENCES

1. Barry, MA.; Behnke, CA; Eastman, A. *Biochem.Pharmacol.* 1990, **40**, 2353-2362
2 . Marks, DI.; Fox, RM.; *Biochem. Pharmacol.* 1991, **42**, 1859-1867
3 . Hickman, JA. *Cancer Metasis. Rev.*,1992, **11**, 121-39
4 . Huschtscha, LI.; Bartier, WA.; Ross, CEA.; Tattersall, MHN. *Br. J. Cancer.* 1996, **73**, 54-60
5. Guchelaar, HJ.; Vermes, I; Koopmans, R.P.; Reutelingsperger, CP., Haanen, C. *Cancer Chemother. Pharmacol.*. 1998, **42**, 77-83
6. Bruserud O., *Int. Immunopharmacol.* 2001, **1**, 2183-2195
7. Bear, L.; Gray, HB.; Rainen, L.; Xhang, Barry YM.; Howard, R.; Serio, G.; Kimball, AP. *Cancer Chemother.* 1975, **5.9**, 611-620
8 . Howard, RA.; Sherwood, E.; Erch, A.; Kimball, AP.; Bear, JL.. *J. Med. Chem.*. 1977, **20**, 943-46
9. Elo, H. *Inorg. Chemica Acta.* 1987, **136**, 133-135
10. Najjar, R.; Santos, FS.; Seidel, W. 1987, *An. Acad. Bras. De Ciências.* **59**:, 13-16
11. Zyngier, S.; Kimura, E.; Najjar, R.. 1989, *J. Med. Biol. Res.* **22**, 397-401
12. Reibscheid, EM.; Zyngier, SB.; Maria, DA.; Mistrone, R.J.; Sinisterra, RD; Couto, LG.; Najjar, R.. *Braz. J. Med. Biol. Res.*, 1994, **27**, 91-94
13. Souza, AR.; Najjar, R ; Glikmanas, S.; Zyngier, S. *J. Inorg. Biochem.* 1996, **64**, 1-5
14. Espósito, BP.; Zyngier, SB; Souza, AR; Najjar, R.. *Metal-Based Drugs,* 1997, **4**, 333-8
15. King, KL.; Cidlowski, J.A *Annu. Rev. Physiol.*, 1998, **60**, 601-617
16 Weller M *J. Neuroloncol.* 1999, **43**, 237-239
17- Nicoletti, I.; Migliorati, G.; Pagliacci, MC.; Grignagni, F.; Riccardi, CA. *J. Immunol. Methods*,1991, **139**,: 271-279
18.Behmer, OA.; Tolosa, E.M.C.; Neto, AGF. *Manual De Técnicas Para Histologia Normal E Patológica.* São Paulo: Edart, 1976..84-86
19. Wyllie, A.H.; Kerr, J.FR.; Currie, A.R. 1980, *Int. Rev. Cytol.*, **68**, 251-306
20. Conover, W.J. *Practical Nonparametric Statistics.* 2°.Ed. New York: John Wiley & Sons, 1980., 229-237
21. Oberhammer, F.; Wilson, JW.; Dive, C,; Morris, ID; Hickman, JA.; Wakeling, AE; Walker, PR.; Sikorska, M. *Embo J.*, 1993, **12**, 3679-84
22. Cheng, J.; Haas, M. *Mol. Cell. Biol.*, 1990, **10**, 5502-5509
23. Lowe, SW.; Rully, HE.; Jacks, T.; Housma, DE *Cell*, 1993, **74**, 957-967
24. Clarke, AR.; Purdie, CA.; Harrison, DJ.; Morris, RG.; Bird, CC.; Hooper, M.L.; Wyllie, AH.. *Nature*, 1993, **362**, 849-852
25. Danova, M.; Giordano, M.; Giuliano, M.; Riccardi, A.. *Leuk. Res.*, 1990, **14**, 417-422
26. Gaidano, G.; Ballerini, P.; Gong, JZ.; Inghirami, G.; Neri, A.; Newcomb, EW.; Magrath, IT.; Knowles DM.; Dalla-Favera, R. *Proc. Natl. Acad. Sci. Usa*,1991, **88**, 5413-5417
27. Gaynon, PS.; Carrel, AL.. *Adv. Exp. Med. Biol.*, 1999, **457**`, 593-605
28.- Roux S; Terouanne B; Defacque H; Vachier I; Loubatiere J; Nicolas JC, *Annal. Biochem*, 1995, **227**, 235-241
29. Fukawa E.; Tanakah.H; Makino Y.; Hirano F.; Akama H.; Kawai S.; Makino I. *Endocr. J.* 1994, **41**, 623-630
30. Helmberg, A.; Auphan, N.; Caelles, C.; Karin, M.. *Embo J.*, 1995, **14**, 452-460
31. Nakao Y; Tsuboi S; Fujita T; Masaoka T; Morikawa S; Watanabe S *Cancer* 1981, **47**, 1812-1817
32. Schster C; Chasserot-Golaz S; Beck G *J. Steroid Biochem.* 1989, **227**, 235-241

**Received: January 9, 2002 – Accepted: January 14, 2002 –**

**Accepted in publishable format: January 24, 2002**

